# Nonhuman primate model in mammary gland biology and neoplasia research

**DOI:** 10.1186/s42826-020-00053-1

**Published:** 2021-01-05

**Authors:** Fitriya N. Dewi, J. Mark Cline

**Affiliations:** 1 Primate Research Center at IPB University, Jl. Lodaya II No.5, Bogor, West Java 16151 Indonesia; 2 Department of Pathology, Section on Comparative Medicine, Wake Forest University School of Medicine, Medical Center Boulevard, Winston-Salem, NC 27157 USA

**Keywords:** Breast cancer, Macaques, Monkey, Breast differentiation, Animal model

## Abstract

Research on breast cancer pathogenesis, prevention and drug development remains an important field as this disease is still one of the leading causes of cancer death worldwide. Nonhuman primates, particularly macaque species, may serve as a highly translational animal model in breast cancer studies due to their similarity with humans in genetics, anatomy, reproductive and endocrine physiology including mammary gland development profile. The use of nonhuman primates in biomedical research, however, requires high ethical standards and an increasing expectation to improve strategies to replace, reduce and refine their use. Here, we discuss some key features of nonhuman primate mammary gland biology relevant to their strengths and limitations as models in studies of breast development and cancer risk.

## Introduction

Breast cancer remains one of the most important diseases worldwide. Despite improvements in screening modalities and therapeutic strategies over the years, breast cancer is still the most commonly diagnosed cancer in women and one of the leading causes of cancer death [1]. Knowledge gaps in the disease pathogenesis need to be filled in order to improve drug development and prevention strategies.

Mammary gland is a unique tissue which only attains its full development postnatally under the influence of growth factors and reproductive hormones. Importantly, reproductive milestones that involve significant breast development, such as menarche, menopause, pregnancy and lactation are considered determinants to the risk of breast cancer [2]. The term “development” refers to structural growth as well as the functional differentiation and regression of the tissue [3]. Stimuli such as steroid hormones, growth hormone, insulin-like growth factor and prolactin play a major part in regulating the differentiation process of the mammary gland in females [4]. In humans, the breast undergoes significant ductal and lobulo-alveolar differentiation at puberty, and this change occurs continuously throughout menstrual cycles and more significantly during pregnancy and lactation. It is therefore important to study the developmental biology of mammary gland pertaining to breast cancer risk, and modification of such risk.

Animal models are imperative to breast cancer-related studies. Various models are available and the decision on which model to select depends on the research questions, such as reproductive window, stage of the disease, immune system and microenvironment, metastatic disease, pre-clinical drug testing, and drug resistance [5]. Mice remain the most used model in breast cancer research, in large part due to the availability of genetically-engineered and xenograft models that allow for studying molecular mechanism of cancer [6]. Nevertheless, nonhuman primates serve as a unique resource for translational research and testing in the field of breast cancer. This review discusses the strength of nonhuman primates, more specifically macaques, as models to study mammary gland in the context of breast development and cancer, and the current challenges and developments in the field.

### Mammary gland development and breast cancer risk

Full development of the mammary gland occurs postnatally. In women, significant elongation and branching of ducts as well as lobular differentiation initiate during puberty under the effect of ovarian hormones [7]. Afterwards, the lobules undergo gradual maturation with increase in the number of terminal ductal and alveolar units per lobule, resulting in a concomitantly larger size. Cyclic expansions of the ducts and lobules occur in response to the hormonal changes during menstrual cycles. More dramatic remodeling of the tissue occurs during pregnancy, lactation and involution, wherein terminal differentiation of the gland can only be attained during pregnancy [8]. Importantly, the degree of lobular differentiation has been inversely related to breast cancer risk [9, 10].

Pregnancy and parity has long been recognized as protective against breast cancer [11]. The protection decreases with the age of the first full-term pregnancy [12], and recent study suggests that the protective effect may not even begin until as many as 30 years later after the birth [13]. Some mechanisms that have been proposed to underlie this protection are: induced differentiation that rids the population of cells that are susceptible to carcinogenesis [14, 15], change in hormone responsiveness [16], sensitization to pro-apoptotic pathways and modified extracellular matrix [17], and altered mammary stem cell fate resulting in persistent changes in the intracellular regulation that governs cell proliferation and response to DNA damage [18].

Adult mammary gland is composed of heterogeneous cells, such as epithelial, fibroblastic, endothelial, immune, and fat cells. Among these various cell types, epithelial cells are arguably the most studied due to their dramatic changes in function and structure throughout the different reproductive stages. During such stages, mammary gland cells proliferate, differentiate or die, resulting in continuous remodeling of the tissue, which indicates the important role of stem and progenitor cells, and the regenerative potential of the mammary epithelium [19]. Epithelial structures of the mammary gland (i.e. ductal, lobular and myoepithelial) initially originate from a common bipotent stem cell, and it has been suggested that a pool of unipotent progenitor cells also exists [19, 20]. The molecular mechanisms governing the differentiation of mammary cells are not well understood, but the process involves bipotent stem cells differentiating into unipotent progenitors, and subsequently to luminal, alveolar or myoepithelial cells. While giving rise to lobuloalveolar epithelial cells, stem and progenitor cells are also considered the cell-of-origin in breast cancer [21]. It has been suggested that breast cancer mainly originates in these undifferentiated cells within the terminal duct of a lobular unit, which are most vulnerable to carcinogenesis [10, 22]. Meta-analysis shows a correlation between the number of normal stem cell divisions and the risk of various cancers [23], which is highly relevant to breast cancer case given the nature of the tissue to undergo cyclic cell division and differentiation. These cells can therefore be an important target in strategizing breast cancer prevention measures as well as therapy.

### Comparative biology of the mammary gland in nonhuman primates and humans

The comparable developmental profile of nonhuman primate breast to humans highlights the importance of this species as a valuable translational model system. The anatomical similarity is supported with a comparable hormonal profile between macaques and humans. Notably, the pattern of estrogen, progesterone, Luteinizing Hormone (LH) and Follicle Stimulating Hormone (FSH) across the ovarian cycle of cynomolgus macaques (*Macaca fascicularis*), rhesus macaques (*Macaca mulatta)* and Japanese macaques (*Macaca fuscata)* are similar to that in women [24, 25]. Duration of the ovarian cycle is 28 to 32 days in macaques with menstrual bleeding ranges between 1 to 8 days (average of 3 to 5 days). During the follicular phase of the cycle, estradiol concentration is approximately 50–200 pg/ml and peaks at 350–380 pg/ml. LH surge occurs in the time of ovulation, showing an approximate value of 700–800 pg/ml [24, 25]. Post-ovulatory progesterone level peaks at 6–10 ng/ml [25, 26]. These values are relatively similar to those reported in women [27]. Macaques also showed comparable sex steroid receptor expression and responses to exogenous hormones [28].

#### Neonatal to puberty

In macaque species, the breast primordia arise bilaterally along the “mammary line”, and small ductal structures grow from the primary bud during fetal development and can be observed as a small rudimentary branching ductal system at birth [29]. The tissue remains rudimentary until the animal reaches puberty, as in humans. The literature on breast development in children is fairly limited. It has been suggested that the duration of mammary gland development from breast growth spurt to menarche is approximately 2–3 years in pubertal girls, and lobule formation occurs within 1 to 2 years after menarche [30, 31].

In macaques, puberty typically occurs at the age of 3–4 years with prominent nipple development preceding regular menstruation [32]. At this stage, ductal and lobular development is also present. We have reported a longitudinal study that observed breast development in cynomolgus macaques during the period spanning 2 years before and after menarche [33, 34], which highlighted the gradual lobular differentiation process during puberty, a profile not seen in rodents [3]. Notably, the lobular differentiation profile in the pubertal macaque breast highly varies between individuals, a phenomenon that is also found in humans.

During the early phase of pubertal breast development in humans and macaques, ductal structures elongate and divide, followed by formation of terminal end buds (TEBs). TEBs contain layers of epithelial cells, particularly 4–8 cell layers of luminal cells and rounded myoepithelial cells [28]. TEBs lead the growth by invading the mammary fat pad and subsequently give rise to new branches and alveolar buds or small ductules. The ductules cluster around a terminal duct and form lobuloalveolar units. This structure is the functional unit that undergoes further differentiation throughout life whereby the ductules increase in number and size. A microarray study indicated that several of the pathways activated during pubertal breast development have been implicated in breast cancer development, which highlights the relevance of pubertal breast development to serve as a model of carcinogenesis [35].

Using the nomenclature developed by Russo et al. [8], lobuloalveolar or lobule units in the macaque breast can also be categorized into types 1, 2 and 3 which consists up to 11, 47 and 80 ductules per unit, respectively. In general, the immature lobule type 1 can be formed as early as 2 years before menarche in the macaque model. The more differentiated lobule type 2 is typically formed closer to menarche, and the biggest change in glandular differentiation can be found around the menarchal transition. The complexity of the lobuloalveolar unit further increases with recurrent menstrual cycles. By early adulthood, the macaque breast is composed mainly of type 1 and type 2 lobules. In addition, type 3 lobules, which typically predominate only when pregnant and lactating, can also be found in lower number [33].

Estrogen and progesterone are important regulators of pubertal breast development. In macaques, estrogen receptor (ER) alpha and beta are expressed in the epithelial cells and often co-expressed in the same cells, with the latter being more abundant [36]. Progesterone receptor (PGR) alpha and beta are also expressed, with PGR alpha being the more abundant [37]. ER and PGR are expressed in TEBs, and proliferation markers are also expressed during periods with high serum estrogen and progestogen concentration [28]. Importantly, studies in the macaque model demonstrated that mammary expression of ER alpha and classic estrogen-regulated markers such as PGR, trefoil factor 1 (TFF1) and growth regulating estrogen receptor binding 1 (GREB1) decreased across the menarchal transition, indicating that estrogen responsiveness in the breast is high in peri-puberty and decreases with adulthood [34]. Adolescence is therefore a critical period of susceptibility to hormonal disruption, and such modulation may have a lasting effect on breast cancer risk later in life. While epidemiological studies have focused on associating pubertal breast development, environmental factors and breast cancer risk [38, 39], macaques may serve as the translational model system to investigate pathways affected at such stages and possible intervention or prevention strategies.

#### Adulthood

Lobule type 1 is the most undifferentiated structure found in young nulliparous women, and is considered the site of origin of ductal carcinomas [40]. In rodents, adult nulliparous (virgin) breast is filled with ductal branching structures but not lobules [3, 4], which is a distinctive difference from humans. The breast of nulliparous women contains mainly type 1 lobules, with moderate and low number of type 2 and 3 lobules, respectively [8]. Adult nulliparous breast of macaques consists primarily of type 2 lobules and the immature type 1 lobules with few type 3 lobules.

Menstrual cycles affect mammary cell proliferation; macaques in the luteal phase show greater proliferative changes in both ducts and lobular epithelial cells [41], a phenomenon similar to that found in women. As cell proliferation is considered a risk factor for carcinogenesis, it has been thought that women with short and numerous cycles may have higher risk of breast cancer and that such effect is likely to be dependent on the roles of progesterone and receptor activator of nuclear factor kappa-B ligand (RANKL) [42].

The breast undergoes extensive growth and differentiation during gestation and lactation. It is important to note, however, that some macaques are seasonal breeder whereas others are not. For example, the two most used macaque species in biomedical research have different features: rhesus macaques (*Macaca mulatta*) are seasonal breeders, whereas cynomolgus macaques (*Macaca fascicularis*) are not. This difference is important when taking into account differences in the breeding pattern and hormones at certain season. The gestational period is approximately 150–175 days in macaque species. A dramatic change occurs in epithelial cell proliferation as well as secretory distention of the ductal and alveolar system under the influence of various hormones, including estrogen, progesterone, chorionic gonadotropin, and prolactin. At this stage, the breast reaches terminal differentiation wherein the gland is predominated by type 3 lobules that have secretory activity. The molecular profile of the breast during pregnancy is markedly different from other life stages. Prolactin is likely to play a key role in alveolar cell development, potentially via GATA Binding Protein 3 (GATA3) and E74 Like ETS Transcription Factor 5 (ELF5). Inversely, there is a distinct pattern of decrease in gene expression for stem cell markers [35].

The lactation period in macaques is typically 1 year and they most often bear one offspring, similar to humans. Afterwards, macaque mammary gland will regress to varying degrees, mostly back to a stage similar to nulliparous breast with a predominance of type 2 lobules [36]. In women, however, it has been reported that the predominant structure of parous breast is type 3 lobules until individuals reach the age of 40 whereby type 2 and 1 lobules will then predominate again [8]. Parity is known to be protective for breast cancer; a recent study in women showed an association with increased risk shortly after childbirth (particularly with later age pregnancy) and decreased risk more than 20 years after childbirth [13]. It is not known if the same applies for macaques, although such an effect is likely given the similarity to humans.

#### Menopause

Menopause in women typically occurs at around 50 years of age, and is preceded by a period of several years of decreasing ovarian function known as the perimenopause period. This stage is marked with irregular cycles leading up to amenorrhea that results from almost complete cessation of ovarian estrogen and progesterone production, a direct consequence of declining numbers of ovarian primordial follicles. This phenomenon leads to various health implications in women for which research often requires the use of nonhuman primates. Menopause occurs naturally in aging macaques (> 22 years of age) also as a result of decline in primordial follicles [43, 44].

In menopausal women, mammary gland regression occurs in both parous and nulliparous women during which type 1 lobules predominate with a concomitant decrease in the number of type 2 and 3 lobules [30]. Further, there is a gradual yet significant loss of mammary epithelial tissue and its functionality whereby older women tend to have fewer lobules or only lobule remnants [45]. Similarly, natural and surgical menopause in macaques results in mammary gland regression, predominated by ductal structures with marked lobular atrophy and little cell proliferative activity [37]. Nevertheless, expression of sex steroid receptors persists, and studies have shown that post-menopausal macaque breast remains responsive to exogenous hormonal stimulation, at least 2 years after surgical menopause [46–49].

Breast cancer risk is known to increase with age in women, although the rate of increase slows at approximately age 50 years, which may be due to breast involution [45]. Importantly, incidental lesions such as cystic change, ductal and lobular hyperplasia, ductal and lobular carcinoma in situ, and invasive ductal carcinoma have been reported in aging macaques [50], highlighting the translational potential of the species for breast cancer research.

### Nonhuman primates as models in breast cancer studies

**Fig. 1:**
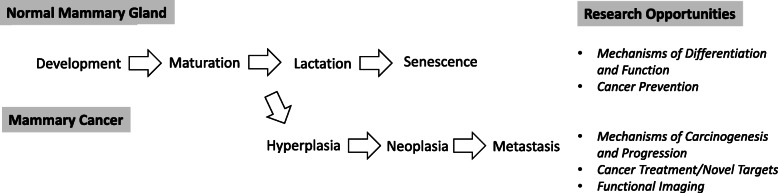
Nonhuman primate research opportunities in the field of breast development and cancer. Nonhuman primates may serve as an important and highly translational model to study various aspects of normal mammary gland development, pre-neoplasia as well as mammary cancer

Several Old World Monkey species have been commonly used in various breast cancer and breast developmental studies such as cynomolgus macaques (*Macaca fascicularis),* rhesus macaques (*Macaca mulatta*), and baboons (*Papio sp.*). More recently, New World Monkeys such as common marmosets (*Callithrix jacchus*) have also been used in mammary studies [51]. The use of various nonhuman primate species in breast cancer and breast developmental studies is summarized in Table 1. Furthermore, Fig. 1 illustrates various research areas in the field of mammary gland biology and cancer wherein the use of nonhuman primates may be of importance.

**Table 1:** Nonhuman primate models in breast development and neoplasia research

Type of Model	Reported Species	References
**Breast Cancer Studies**		
Spontaneous mammary neoplasia	*Macaca mulatta*	[50, 52–54]
	*Macaca fascicularis*	
	*Papio sp.*	
	*Chlorocebus aethiops*	
Chemically-induced mammary neoplasia	*Macaca mulatta*	[55]
	*Macaca fascicularis*	
	*Chlorocebus aethiops*	
**Breast Developmental and/or Cancer Risk Studies**		
HRT or SERM exposure	*Macaca fascicularis*	[36, 46–49, 56–59]
Dietary exposure	*Macaca fascicularis*	[33, 34, 48, 49, 60]
EDC	*Macaca mulatta*	[61]
**Other Applications**		
Breast profiling at specific developmental stages to model carcinogenesis	*Macaca fascicularis*	[28, 33–35, 41]
	*Macaca mulatta*	
Ex vivo mammary culture model	*Macaca mulatta*	[51, 62, 63]
	*Macaca fascicularis*	
	*Papio hamadryas anubis*	
	*Callithrix jacchus*	

*HRT* Hormone replacement therapy, *SERM* Selective estrogen receptor modulator, *EDC* Endocrine disrupting chemicals

#### Spontaneous mammary neoplasia

**Fig. 2:**
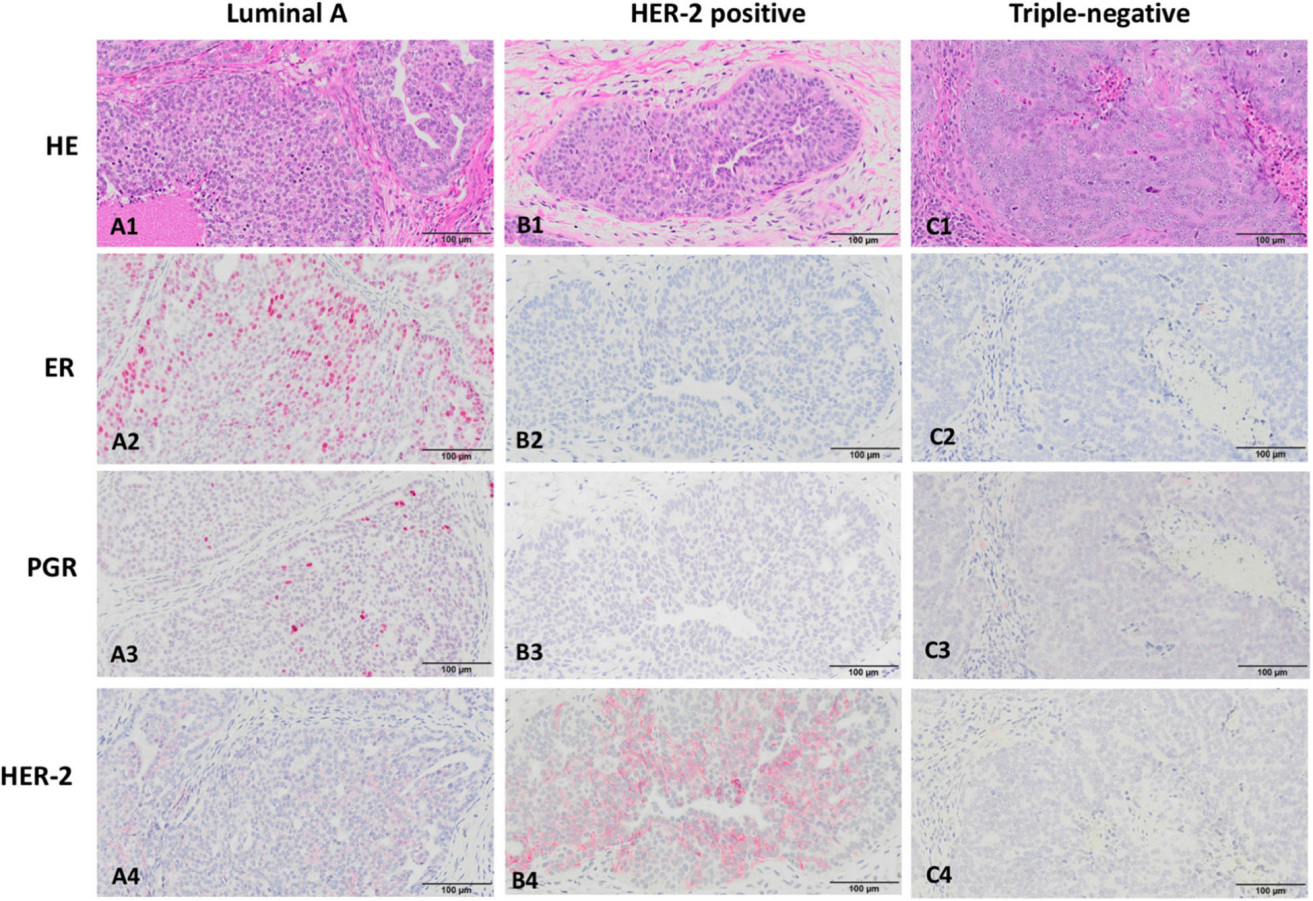
Histological profile of breast cancer in macaques. The morphology (Hematoxylin-Eosin stained; HE) and immunohistochemical staining for estrogen receptor (ER) alpha, progesterone receptor (PGR) and human epidermal growth factor receptor 2 (HER-2) are shown to indicate a Luminal A type (A1-A4), HER-2 positive (B1-B4), and triple-negative (C1-C4) breast cancer, which are similar to those in humans. Strong positive nuclear staining of ER alpha (A2) and PGR (A3) are shown in Luminal A type; HER-2 positive breast cancer shows cytoplasmic overexpression of HER-2 (B4); and ER, PGR and HER-2 are not expressed in triple-negative breast cancer (C2-C4). Bars = 100 μm

Cynomolgus and rhesus macaques show spontaneous mammary ductal hyperplastic and neoplastic lesions in older captive animals, with an estimated lifetime incidence of carcinoma (in situ and invasive) to be about 6% [50]. Importantly, morphologic characteristics of the lesion are similar to that in humans, allowing for categorization of neoplasia using human terminology (e.g. histological grading system). The majority of breast carcinomas in macaques are of “Luminal A” type, expressing both ER and PGR; HER-2 overexpressing tumors have also been reported, and some carcinomas do not express ER, PGR and HER-2, known as “triple negative” as in humans (Fig. 2). Tumors may also progress into metastatic disease, with the typical sites being axillary lymph nodes, lungs, liver and kidneys. Spontaneous mammary gland carcinomas reported in rhesus macaques are not limited to older animals, and viral etiology has been proposed alongside other factors such as hormones and irradiation [52]. In baboons, spontaneous mammary gland carcinoma of ductal origin has been reported with features similar to that in humans [53]. Although less relevant in biomedical research use, prosimians such as lemurs and galagos have also been reported to show spontaneous mammary gland neoplasia [64]. Despite a known incidence, invasive breast cancer cases in nonhuman primates are still considered rare and may be under-observed or underreported. Some factors that may contribute to this rarity are the fact that majority of macaques are not kept until their natural post-menopausal ages, the lack of nulliparous macaques available to be studied, and that macaques live a relatively “healthier” life (i.e. non-obese animals fed with a nutritionally-balanced diet).

#### Induced model of mammary neoplasia

Induction of breast cancer in macaques is generally considered to be impractical; such induced tumors would likely occur across a period of years. Although chemical carcinogenesis studies have been done in nonhuman primates [55], and breast cancers have occurred in carcinogen-treated macaques, such tumors were rare and thus may reflect only the spontaneous background incidence of breast cancer. It is likely that nonhuman primates’ response to carcinogen is distinctive from that of rodents. Notably, the use of dimethylbenzanthracene (DMBA), a mammary carcinogen typically used in rat cancer studies, did not elicit cancer in stump-tailed macaques (*Macaca arctoides)* following a 4 year observation period [65].

#### Breast cancer risk model

Up to now, there is no reported study utilizing monkey cancer patients as subjects for intervention. The challenge in doing so may be related to the heterogeneity of lesions observed in monkeys as compared to that in induced rat models or genetically-engineered mice [54]. The majority of the macaque studies on the mammary gland have been related to breast cancer risk instead of *actual* breast cancer incidence. Similar to human breast, the mammary gland of nonhuman primates is also responsive to reproductive hormones and growth factors. Consequently, receptors such as ER, PGR and proliferation markers such as Ki67 have been used as breast cancer biomarkers to predict risk.

One of the landmark examples of the translational importance of monkey breast study was the Hormone Replacement Therapy (HRT) study in cynomolgus macaques reported by Cline et al. [56]. This study suggested higher risk of breast cancer with treatment of estrogen plus progestin, a finding that was later found to be true in Women’s Health Initiative Study and resulted in termination of the trial [66]. Over the years, cynomolgus macaques have been used in HRT or selective estrogen receptor modulator (SERM) studies in regards to breast cancer risk [47, 57–59, 67]. Another report relevant to this topic was an earlier review of 17 contraceptive steroid studies performed in macaques and baboons, which suggested the risk of spontaneous tumors although with low incidence [68]. In addition, macaques have also been used in studying exposure to various dietary compounds (e.g. Western, Mediterranean, soy-based) or endocrine disrupting chemical during specific life stages such as in-utero, puberty and adulthood to evaluate the effects on breast development and subsequent breast cancer risk [33, 34, 60, 61].

#### The future of utilizing monkey breast cancer patients

Studies of macaques with breast cancer have been primarily descriptive. The initial studies showed that the 3 main breast cancer subtypes seen in women (Luminal A/B, HER-2 overexpressing, and basal/triple negative) occur in similar proportions in macaques [50]. These cancers provide an opportunity for testing novel hormonal therapies targeting ER/PGR positive tumors, for example aromatase inhibitors to decrease estrogen production, or SERMs which antagonize the ER alpha pathways in the breast [47].

Increasingly, immunotherapy is used to target cancers. Targeting of HER-2 overexpressing cancers in women was one of the first success stories in activating the immune response against cancer [69]. The greatest remaining challenge for breast cancer treatment is the triple-negative breast cancer (TNBC) or basal type, for which specific therapeutic strategies are lacking. There is some evidence that immune checkpoint inhibitors (ICI) may improve survival in human TNBC [70], but no studies of ICI treatment have been published in macaques. Targeting of tumor-associated fibroplasia in breast cancer has been demonstrated in preliminary studies [71], and additional studies using this approach are in progress (Cline, unpublished data). The close molecular similarities between humans and macaques, and the larger size of macaques compared to rodents, allows the conduct of preclinical proof of concept studies using positron emission tomography ligands designed to bind to human proteins. This concept has been demonstrated for naturally-occurring colon cancer in rhesus macaques [72], and studies of naturally-occurring breast cancers in macaques would also have high translational value.

### Advancing 3Rs in the use of nonhuman primates for breast cancer-related studies

In the United States and more recently in most countries in Asia, all research using Nonhuman Primates must adhere to various laws and regulations, including a mandatory oversight by ethics committee (typically an Institutional Animal Care and Use Committee; IACUC). There is a constant requirement to minimize animal use and suffering in adherence to the 3Rs (Replacement, Reduction, Refinement) principle by Russell and Burch [73] but some cultural and practical barriers to implement methods that can replace, reduce and refine animal use remain, and apply to vertebrate species beyond monkeys. Importantly, there is arguably a greater expectation to overcome them in the case of nonhuman primates [74]. Nonhuman primates have significant cognitive capacity and complex social behavior, often raising issues in justifying the rationale of their use in biomedical research [75]. Therefore, use of nonhuman primates come with increasing ethical considerations and higher public scrutiny due to such higher sentience of the animal and greater societal concern. In regards to such expectations, it is important to always provide a solid justification on why nonhuman primates use is irreplaceable for a proposed study, and therefore one must carefully consider every aspect of the 3Rs on a greater length.

In implementing Reduction, sample size in a nonhuman primate study must be kept at minimum and requires a solid sample size calculation, relatively similar to that for other animal studies. Pilot study is often useful to provide preliminary data to allow for a more precise sample size calculation based on statistical power. Refinement must be carefully strategized due to higher degree of sentience of nonhuman primates. It is important to have adequate environmental enrichment program designed based on the behavioral science of the species in order to promote their psychological well-being. Ideally, the program can be managed and performed by dedicated enrichment team or personnel [76], and the plans should take into consideration the unique behavioral patterns of each individual species. An animal training program with positive interaction with humans is also encouraged to allow for a more refined approach in experimental procedures [77]. Another common refinement strategy is the use of advanced imaging modalities, which has become a popular choice in cancer research as it can allow for less invasive procedures and less number of animals.

For Replacement consideration, one must always set a clear scientific question and objectives in order to identify whether nonhuman primate use is justified. Primate models are highly suitable for certain research fields such as atherosclerosis, behavior, cognition, neuroscience, aging, genetics, and reproduction [75]. Macaques in particular are often chosen as model in drug safety assessment due to their close genetic homology to humans (~ 93%) [78]. Nevertheless, *Replacement* (both in the definition of “relative replacement” and “absolute replacement”) remains the constant request or question by the IACUC in every use of nonhuman primates. The use of “lower” animal model such as rats and mice, especially genetically-modified mice has become the significant replacement strategy in breast cancer studies [79]. However, such a strategy may still be unsuitable when the scientific question involves a particular phenomenon absent in non-primate models. In the context of the mammary gland, for example, rodents are known to undergo prominent ductal elongation but not mammary lobular growth prior to pregnancy [3, 80]. In humans, puberty milestone such as thelarche (i.e. the onset of breast development) have been associated with breast cancer risk [81]. Nonhuman primates are arguably still the more translational model when it comes to studying lobular development in puberty up to adult nulliparity [33, 34]. Pubertal breast can also be a model system for studying epithelial cell morphogenesis and cancer susceptibility [82], and modulating breast differentiation during this life stage may have a long term impact on breast cancer risk. This important developmental window is highly important yet relatively understudied, and nonhuman primate models are needed to fill the knowledge gap. Several genetically-engineered mouse models may serve as alternatives to study pubertal mammary gland, but the focus may be limited to ductal growth, branching and TEBs [82].

The other biological phenomena that are difficult to mimic in non-primate model are menstruation and menopause. Menarche and menopause are established risk factors of breast cancer, and therefore studying the mammary gland during those stages is essential. Also, primates have a comparatively long life-span of the ovarian corpus luteum, irrespective of whether conception occurs, and various macaques have a highly similar ovarian cycle to that of women [24, 25]. It has been thought that the cyclic stimulation of ovarian hormones affects breast cell proliferation and subsequently the risk of cancer [42, 81]. Rodents have estrus cycle as opposed to a menstrual cycle, and estropause instead of menopause, which is a significant limitation in research specific to menstrual cycle and risk to breast cancer. Importantly, however, recent evidence showed that the spiny mouse (*Acomys cahirinus*) is the first rodent species that exhibited menstruation with endometrium profile similar to human menstrual shedding [83]. It is unknown, however, if such resemblance also applies to other reproductive systems such as the mammary gland.

A common Replacement strategy is the use of in vitro models such as established cell culture lines. In terms of “normal” or non-cancer cell line, non-primate mammary cells are greatly utilized, but some limitations in the translational aspects such as genetics and certain hormone receptors still need to be considered. While human-derived normal cell lines are available, they are mostly limited to those of post-menopausal backgrounds, and often from individuals with benign breast disease. Furthermore, it is ethically restricted to acquire normal human breast tissue during specific developmental stages. The use of nonhuman primate-derived tissue serves to bridge such gap. Mammary gland tissue of nonhuman primates such as common marmoset [51] and rhesus macaques [63] have been used in mammosphere culture studies to enrich mammary stem cells. As more refined approaches are needed to acquire such tissue, our recent study of cynomolgus macaque mammosphere culture utilized small samples of mammary tissue taken by biopsy instead of at necropsy [62], suggesting the feasibility of non-terminal study using a limited amount of tissue as the source for cell culture.

## Conclusions

Mammary gland development is a continuous process regulated by hormones and growth factors and may determine breast cancer risk. Studies are required to understand the mechanisms of breast development and carcinogenesis in order to develop effective disease prevention and therapeutic strategies. Nonhuman primates, particularly macaques have a comparable breast development profile to humans, including in aspects that are lacking in other animal models. It is important to understand these key features to consider the appropriateness of macaques as model in breast cancer-related studies.

## Data Availability

Not applicable for review article.

## Abbreviations

3Rs: Replacement, Reduction, Refinement
DMBA: Dimethylbenzanthracene
EDC: Endocrine disrupting chemical
ELF5: E74 Like ETS transcription factor 5
ER: Estrogen receptor
FSH: Follicle stimulating hormone
GATA3: GATA binding protein 3
GREB1: Growth regulating estrogen receptor binding 1
HER-2: Human epidermal growth factor receptor 2
HRT: Hormone replacement therapy
IACUC: Institutional Animal Care and Use Committee
ICI: Immune checkpoint inhibitors
LH: Luteinizing hormone
PGR: Progesterone receptor
RANKL: Receptor activator of nuclear factor kappa-B ligand
SERM: Selective estrogen receptor modulator
TEB: Terminal end bud
TFF1: Trefoil factor 1
TNBC: Triple-negative breast cancer
